# Supplementary material for: Expression Analysis of a Novel Oxidoreductase Glutaredoxin 2 in Black Tiger Shrimp, Penaeus monodon
Source: Antioxidants (Basel). 2022 Sep 20;11(10):1857. doi: 10.3390/antiox11101857 (PMC9598912; doi:10.3390/antiox11101857)
Supplement: Supplementary file 1 [file antioxidants-11-01857-s001.zip › Supplementary Materials-Figure.pdf]

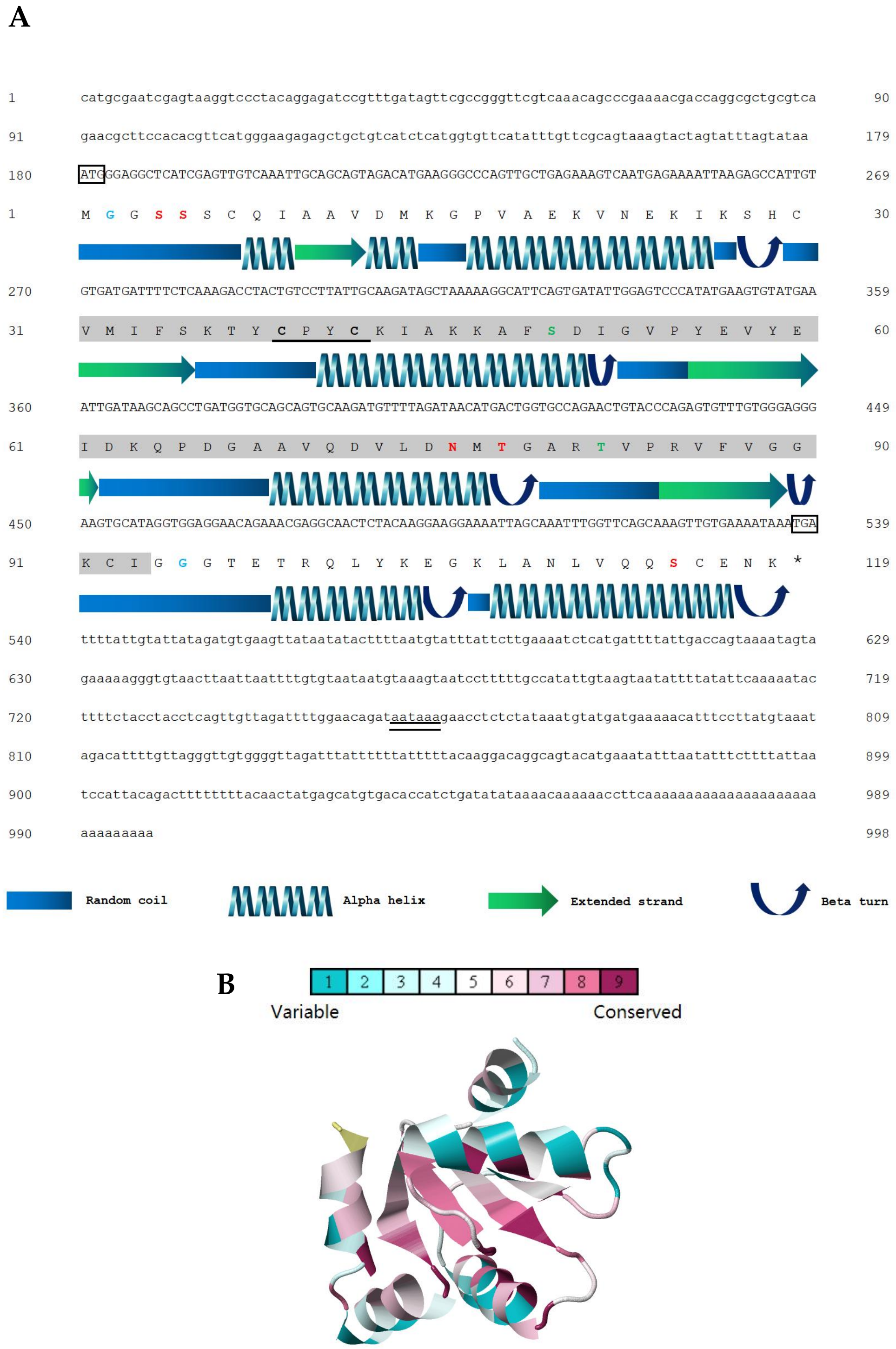

Figure S1. (A) The sequence of nucleotides, the deduced sequence of amino acids, and the putative secondary structure of *PmGrx2*. The initiation codon "ATG" and the termination codon "TAA" are marked in the rectangular box. The poly adenylation signal sequence (AATAAA) is marked using double underlined. The grey region indicates the predicted Grx structural domain. The active center sequence (C-P-Y-C) of the Grx domains is marked using single underlined. Cys residues are marked in bold. The phosphorylation sites, glycosylation sites, and N-myristoylation sites are highlighted in green, red, and blue, respectively. The legend of the predicted secondary structure is annotated at the bottom of the image. (B) The putative tertiary structure of *PmGrx2*.

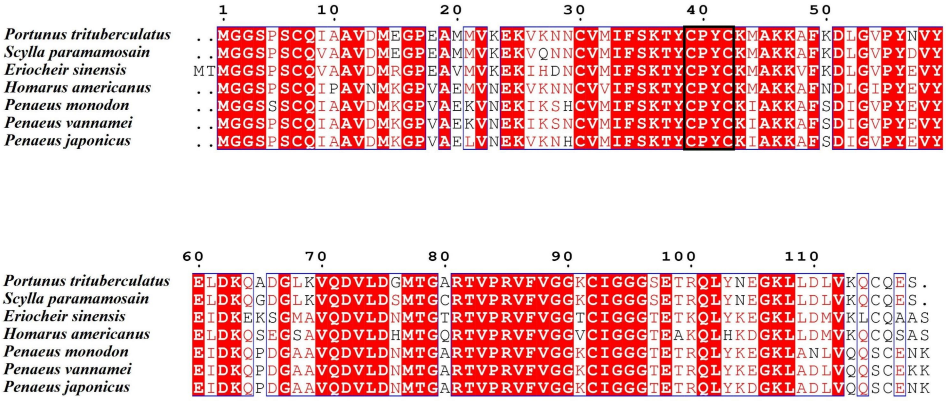

Figure S2. Multiple sequence alignment of Grx2 from seven crustacean species. Red regions indicate conserved amino acid residues. Red letters indicate similar residues. Conserved active sites C-P-Y-C are highlighted in a black box. The results of the amino acid sequence counts are shown at the top of each row.

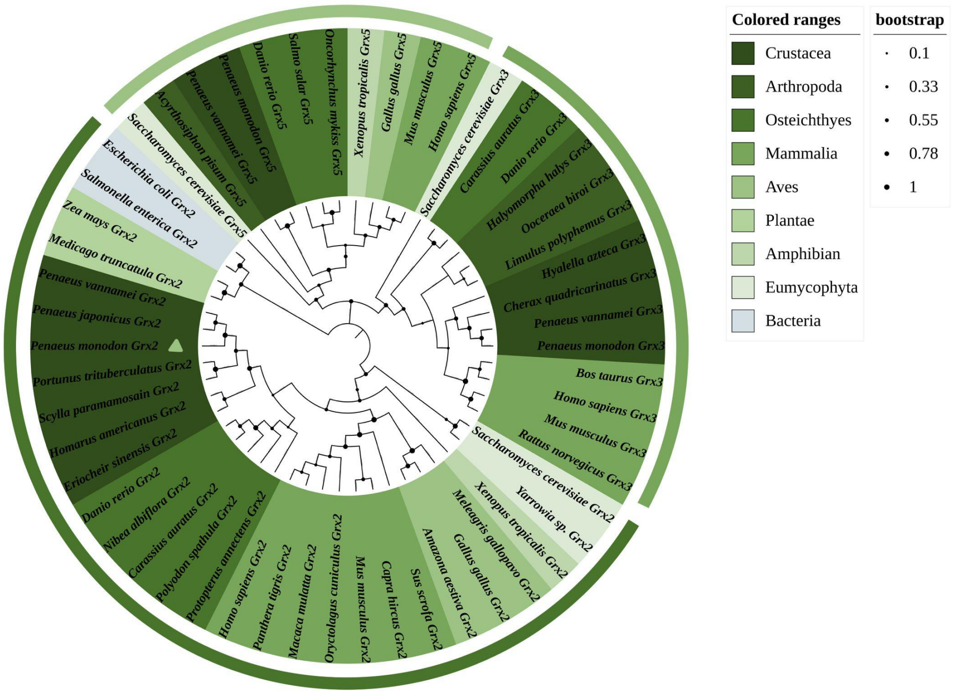

Figure S3. Phylogenetic tree of Grx2, Grx3 and Grx5 based on amino acid sequences. The corresponding bootstrap support values for each branch are indicated by dark green dots. The size of the dark green dots and the corresponding values are shown on the right side of the image. The corresponding species classification in different colours is shown on the right side of the image. *PmGrx2* is marked with triangles.
