# Supplementary material for: Expression Analysis of a Novel Oxidoreductase Glutaredoxin 2 in Black Tiger Shrimp, Penaeus monodon
Source: Antioxidants (Basel). 2022 Sep 20;11(10):1857. doi: 10.3390/antiox11101857 (PMC9598912; doi:10.3390/antiox11101857)
Supplement: Supplementary file 1 [file antioxidants-11-01857-s001.zip › Supplementary Materials-Table.pdf]

Supplementary materials-Table

Table S1. Primers used in cDNA cloning.

Forward primer 5′-3′ sequences	Reverse primer 5′-3′ sequences	Purpose
GCTGCTGTCATCTCATGGTGTTCA	TCCTTCCTTGTAGAGTTGCCTCGTT	
ATGAAGGGCCCAGTTGCT	CTGAACCAAATTTGCTAATTTTC	*PmGrx2*-ORF Validation
GCATAGGTGGAGGAACAG	CTAACAACTGAGGTAGGTAGAA	

Table S2. Primers used in RACE.

Primer Name	5′-3′ sequences
UPM Long primer	CTAATACGACTCACTATAGGGCAAGCAGTGGTATCAACGCAGAGT
UPM Short primer	CTAATACGACTCACTATAGGGC
NUP	AAGCAGTGGTATCAACGCAGAGT
PmGrx2-3′ GSP	TGATAAGCAGCCTGATGGTG
PmGrx2-5′ GSP	CTAACAACTGAGGTAGGTAGA

Table S3. Primers used in qRT-PCR experiments.

Forward primer 5′-3′ sequences	Reverse primer 5′-3′ sequences	Purpose
AAGCCAGGTATGGTTGTCAACTTT	CGTGGTGCATCTCCACAGACT	*PmEF-1α* qRT-PCR primers
TGATAAGCAGCCTGATGGTG	GCCTCGTTTCTGTTCCTCCA	*PmGrx2* qRT-PCR primers
TCCTCCGTCCTTCGTGTCTCTTCCT	ACCAGGTGGCGTAGAAGTCGATGAC	*PmTrx* qRT-PCR primers
TACCCTTTGGATTTCACCTTTGTC	ATTGATTGTTACCTGACGGAGATT	*PmPrx1* qRT-PCR primers
CGAGGATTTGCTGTGAAGTTTT	ATGAAGGAAGGGAATAGAATAGGAT	*PmCAT* qRT-PCR primers
GGTGACATCGAGAAGGGCAAGAAGA	CCTTGGACTTGTTGGCGTCTGTGTA	*PmCYC* qRT-PCR primers
ATGGAGCCCTGTATTGAG	GTGGATGGAGAACTGGAA	*PmIAP* qRT-PCR primers

Table S4. Primers used in dsRNA synthesis.

Forward primer 5′-3′ sequences	Reverse primer 5′-3′ sequences	Purpose
ATGGTGAGCAAGGGCGAGGAG	TCAAAGATCTACCATGTACAGCTCGT	dsGFP Synthesis
TAATACGACTCACTATAGGATGGTGAGCAAGGGCGAGGAG	TAATACGACTCACTATAGGTCAAAGATCTACCATGTACAGCTCGT	dsGFP Synthesis (T7)
AGGCTCATCGAGTTGTCAAATTGC	TCCTTCCTTGTAGAGTTGCCTCGT	dsPmGrx2 Synthesis
TAATACGACTCACTATAGGAGGCTCATCGAGTTGTCAAATTGC	TAATACGACTCACTATAGGTCCTTCCTTGTAGAGTTGCCTCGT	dsPmGrx2 Synthesis (T7)

Table S5. Primers used in exons amplification of *PmGrx2*.

Forward primer 5′-3′ sequences	Reverse primer 5′-3′ sequences	Purpose
TGAAAATGCCCCAATAGTT	ATACTCTTACCTTTTTAGCTATCTT	Exon1 amplification of *PmGrx2*
TGATTTGTTACACTGACAGGCAT	AAGGCTACACCTGTTCCCTG	Exon2 amplification of *PmGrx2*
ACCTTGTTTCCCCAGTGAT	AGTTACACCCTTTTTCTACTATTTT	Exon3 amplification of *PmGrx2*
AAACTGAAATAACATTCCATTG	AAGGATTACTTTACATGATTACAC	Exon4 amplification of *PmGrx2*

Table S6. Accession numbers were used the alignment and phylogenic tree in this study.

Accession number	Scientific name
KQK78544.1	*Amazona aestiva*
AK341132.1	*Acyrthosiphon pisum*
ADY47130.1	*Ascaris suum*
PSN36174.1	*Blattella germanica*
NP_001030273.1	*Bos taurus*
XP_026053840.1	*Carassius auratus*
XP_026078075.1	*Carassius auratus*
XP_005690513.1	*Capra hircus*
KAG0717517.1	*Chionoecetes opilio*
AEL23128.1	*Cherax quadricarinatus*
WP_190627512.1	*Cyanobacteria*
NP_001005950.1	*Danio rerio*
NP_001002404.1	*Danio rerio*
BC059659.1	*Danio rerio*
OAF97418.1	*Escherichia coli*
SYX47894.1	*Escherichia coli*
AXM05418.1	*Eriocheir sinensis*
XP_422200.3	*Gallus gallus*
AJ720261.1	*Gallus gallus*
XP_042205294.1	*Homarus americanus*
XP_042230596.1	*Homarus americanus*
KAA0195653.1	*Hyalella azteca*
AAH05289.1	*Homo sapiens*
AAH28113.1	*Homo sapiens*
AB223038.1	*Homo sapiens*
XP_013786644.1	*Limulus polyphemus*
XP_010713979.1	*Meleagris gallopavo*
NP_001253327.1	*Macaca mulatta*
NP_001033681.1	*Mus musculus*
NP_075629.2	*Mus musculus*
AK013761.1	*Mus musculus*
AES71315.2	*Medicago truncatula*
KAG8003369.1	*Nibea albiflora*
EZA62809.1	*Ooceraea biroi*
XP_008266946.1	*Oryctolagus cuniculus*

BT073697.1	*Oncorhynchus mykiss*
XP_043941258.1	*Protopterus annectens*
MZ827442	*Penaeus monodon*
XP_042879398.1	*Penaeus japonicus*
XP_042868605.1	*Penaeus japonicus*
XP_041125683.1	*Polyodon spathula*
XP_042832948.1	*Panthera tigris*
XP_045130901.1	*Portunus trituberculatus*
MPC08028.1	*Portunus trituberculatus*
XP_027229698.1	*Penaeus vannamei*
XP_027209771.1	*Penaeus vannamei*
XP_027214062.1	*Penaeus vannamei*
XM_027358261.1	*Penaeus vannamei*
AAH86381.1	*Rattus norvegicus*
GFP69650.1	*Saccharomyces cerevisiae*
GFP69319.1	*Saccharomyces cerevisiae*
NM_001183873.1	*Saccharomyces cerevisiae*
AGS29206.1	*Salmonella enterica*
UHY14109.1	*Scylla paramamosain*
BT048738.1	*Salmo salar*
NP_001230646.1	*Sus scrofa*
QCE13504.1	*Vigna unguiculata*
NP_001016637.1	*Xenopus tropicalis*
BC075374.1	*Xenopus tropicalis*
KAG5373225.1	*Yarrowia sp.*
NP_001151051.2	*Zea mays*

Table S7. Homology of *PmGrx2* with Grx2 of other species.

Species	Identity %	Similarity %
Penaeus vannamei	96.64	98
Penaeus japonicus	94.96	98
Portunus trituberculatus	77.97	87
Eriocheir sinensis	77.88	86
Homarus americanus	76.32	92
Scylla paramamosain	75.42	88

Table S8. Names and Websites of the analysis software.

Software Name	Software Website
ORF finder	https://www.ncbi.nlm.nih.gov/orffinder
BLAST	https://blast.ncbi.nlm.nih.gov/Blast.cgi?CMD=Web&PAGE_TYPE=BlastHome
SMART	http://smar t.embl-heidelberg.de/
SignalP-5.0 Server	http://www.cbs.dtu.dk/services/SignalP/
YinOYang 1.2 Server	http://www.cbs.dtu.dk/services/YinOYang/
NetNGlyc 1.0 Server	http://www.cbs.dtu.dk/services/NetNGlyc/

NetPhos 3.1 Server	http://www.cbs.dtu.dk/services/NetPhos/
Motif Scan	https://myhits.sib.swiss/cgi-bin/motif_scan
SOPMA	https://npsa-prabi.ibcp.fr/cgi-bin/npsa_automat.pl?page=/NPSA/npsa_sopma.html
ConSurf	https://consurf.tau.ac.il/overview.php
Expasy	https://web.expasy.org/compute_pi/
NCBI	https://www.ncbi.nlm.nih.gov/
STRING	https://cn.string-db.org/
